# Robotic exoskeleton-assisted walking rehabilitation for stroke patients: a bibliometric and visual analysis

**DOI:** 10.3389/fbioe.2024.1391322

**Published:** 2024-05-17

**Authors:** Shuangshuang Wen, Ruina Huang, Lu Liu, Yan Zheng, Hegao Yu

**Affiliations:** ^1^ The Eighth Affiliated Hospital, Sun Yat-sen University, Shenzhen, China; ^2^ Shenzhen Health Capacity Building and Continuing Education Center, Shenzhen, China

**Keywords:** robotic exoskeleton, rehabilitation, stroke, walking, bibliometric

## Abstract

**Objective:**

This study aimed to conduct a bibliometric analysis of the literature on exoskeleton robot assisted walking rehabilitation for stroke patients in the Web of Science Core Collection over the past decade.

**Method:**

Retrieved literature on exoskeleton robot assisted gait training for stroke hemiplegic patients from the Web of Science Core Collection from 1 January 2014 to 31 January 2024. The search method was topic search, and the types of documents were “article, meeting abstract, review article, early access.” CiteSpace was used to analyze the search results from countries, institutions, keywords, cited references and cited authors.

**Result:**

A total of 1,349 articles were retrieved, and 1,034 were ultimately included for visualization analysis. The annual publication volume showed an upward trend, with countries, institutions, and authors from Europe and America in a leading position. The core literature was also published by authors from European and American countries. The keywords were divided into 8 clusters: # 0 soft robotic exit, # 1 robot assisted gain training, # 2 multiple scales, # 3 magnetic rheological brake, # 4 test retest reliability, # 5 electromechanical assisted training, # 6 cerebra salary, and # 7 slow gain. The early research direction focused on the development of exoskeleton robots, verifying their reliability and feasibility. Later, the focus was on the combination of exoskeleton robot with machine learning and other technologies, rehabilitation costs, and patient quality of life.

**Conclusion:**

This study provides a visual display of the research status, development trends, and research hotspots, which helps researchers in this field to grasp the research hotspots and choose future research directions.

## 1 Introduction

Stroke is the third leading cause of disability globally (Feigin et al., 2022). Although the age-standardized mortality rate for stroke is declining (Krishnamurthi et al., 2020), the incidence remains high, with over 12.2 million new cases occurring worldwide annually (Feigin et al., 2021). Hemiplegia is a primary challenge faced by affected individuals and their caregivers, bringing a substantial burden on patients and their families. Post-stroke patients require rehabilitation to regain ambulatory and balance capabilities, essential for maintaining normal activity levels (Warutkar et al., 2022). However, data indicates that 70% of individuals experience reduced walking speed and capacity post-stroke, with 20% becoming reliant on wheelchairs, which significantly impeding their independence in daily living (Mehrholz et al., 2020; Kim and Kim, 2022). Traditional rehabilitation is delivered by therapists or nurses, but the rising incidence of stroke has led to a shortage of qualified professionals (Mehrholz et al., 2020). The advent of rehabilitation robotics offers a novel solution to this issue, as these devices can assist in performing repetitive and standardized rehabilitation tasks (Riener et al., 2005), thereby alleviating the burden on rehabilitation workers (Yang et al., 2023). In recent years, due to advancements in chemical material sciences, control technologies, and the integration of artificial intelligence, there has been a rapid development of exoskeletal robotic devices (Yi and Yubing, 2024). These devices have emerged as groundbreaking in the adjunctive therapy of post-stroke hemiparesis, facilitating the reclamation of ambulatory abilities and gait balance (Rodríguez-Fernández et al., 2021). Contrasting with end-effectors, robotic exoskeleton (RE) are wearable robotic units that are affixed to an individual’s limbs, providing either a substitution or enhancement of limbs movement. In the context of ambulation therapy for patients with hemiparesis, these RE emulate normative walking patterns to aid in the movement of the lower limbs, targeting the correction of gait or assistance in walking (Pons, 2008). This modality not only promotes greater freedom of movement for the patients but also actively engages their motor awareness, fostering a conducive environment for motor learning.

Current research on RE in the context of gait rehabilitation therapy for stroke patients is bifurcated into two main domains: technological development and therapeutic efficacy. Technological investigations are concentrated on the biomimetic design of exoskeletal mechanisms, the detection of movement intention, and the motor control within human-machine hybrid systems. The efficacy dimension focuses on the assessment of patient gait restoration, the consequent effects on neuroplasticity, and the overall impact on the patients’ self-care capabilities and quality of life (Koenig et al., 2011; Loro et al., 2023; Pournajaf et al., 2023; Yang et al., 2023). Some scholars have provided comprehensive reviews on the effectiveness of RE in rehabilitative treatments for stroke survivors (Yang et al., 2023). However, traditional reviews or systematic reviews prioritize the synthesis and critical appraisal of literature, aiming to address specific questions within a given field (Yu et al., 2017). Bibliometrics is an interdisciplinary field that employs statistical methods to depict the characteristics and relationships of existing publications, active scholars, research institutions, research topics, or keywords within a research domain, as to as highlighting gaps in the field and predicte hotspots and trends over time (Hu et al., 2020; Donthu et al., 2021; Ninkov et al., 2022).

There has been a significant increase in bibliometric studies on post-stroke patients in the past 2 years, including patient rehabilitation and treatment (Dong et al., 2022; Harjpal et al., 2022; Uivarosan et al., 2022; Zuccon et al., 2022; Ogihara et al., 2023; Wu et al., 2023; Cheng and Yu, 2024), dysphagia (Xu et al., 2023), cognitive impairments (Chi et al., 2023), and pain (Li et al., 2022). However, studies on rehabilitation robots are few and far between. Giacomo Zuccon and other scholars had conducted bibliometric analyses on the technologies of post-stroke rehabilitation robots over the last 20 years, suggesting an increasing focus on the application of robots in the early-stage lower limb training of stroke patients (Zuccon et al., 2022). Diana Uivarosan and others presented relevant data from journals, authors, countries and institutions, and analyzed literature on the application of robots in rehabilitation for stroke patients (Zuccon et al., 2022). There is an increasing amount of research on the technological and application aspects of RE assisting in gait therapy for stroke patients, yet bibliometric studies in this area are still unseen. Therefore, this study conductd a bibliometric analysis on the research of exoskeleton robots in gait rehabilitation of stroke patients over the past decade, which was crucial for understanding the development of this field and selecting future research directions.

## 2 Data and methods

### 2.1 Data source and search strategy

A literature search on studies related to exoskeleton robot-assisted gait training after stroke was conducted in the Web of Science Core Collection database. The search strategy was topic search, covering the period from 1 January 2014 to 31 January 2024. The types of documents searched included “article, meeting abstract, review article, early access". The search strategy was as follows:#1 TS = (Exoskeleton robot OR rehabilitation robot OR robotic exoskeleton OR robot-assisted); #2 TS = (stroke OR apoplexy OR cerebrovascular accident OR cerebral hemorrhage OR hematencephalic OR encephalorrhagia OR cerebral ischemia); #3 TS = (walk* OR gait OR ambulation OR locomotion OR leg* OR lower limb* OR lower extremit*); #4 = (#3 AND #2 AND #1).

### 2.2 Analysis tools

In this study, we utilized the CiteSpace software developed by Professor Chaomei Chen for bibliometric visualization analysis. CiteSpace is among the leading tools for creating knowledge maps, capable of delineating the current state and predicting future research prospects and hotspots in a given field. By employing co-citation analysis and the Path Finder network algorithm, CiteSpace quantitatively analyzes literature. It conducts a complex network analysis that is diversified, time-phased, and dynamic by tracing the formation, accumulation, diffusion, transformation, and evolutionary paths of citation clusters and their knowledge inflection points. CiteSpace explores the current status, development trends, key points, research hotspots, research frontiers, and evolution process of a scientific field, and judge the development trends in the furture.

### 2.3 Data extraction

**FIGURE 1:**
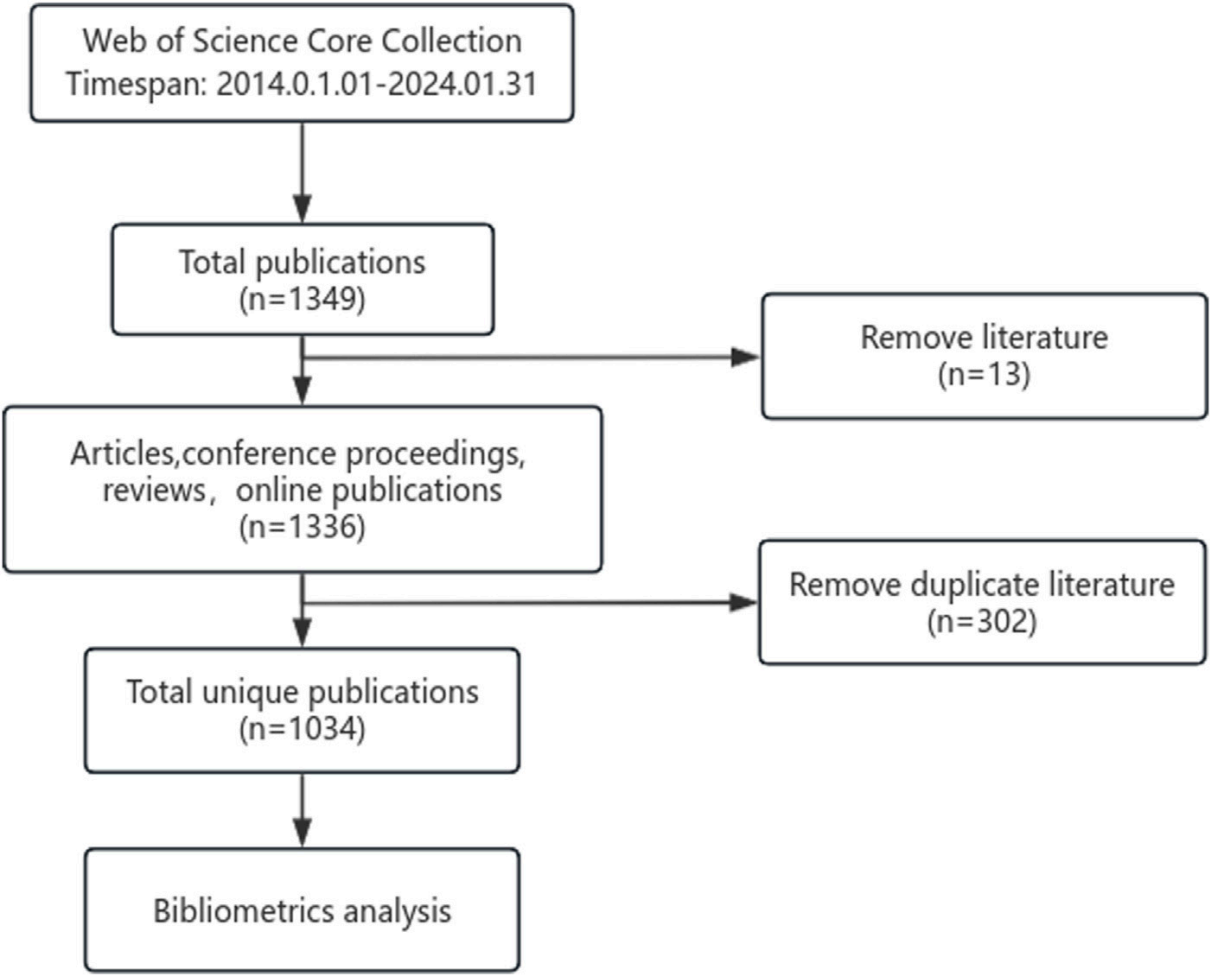
Flow chart of literature selection.

We employed CiteSpace 6.3. R1 software for bibliometric analysis of literature retrieved from the Web of Science database. Literature from the Web of Science was exported in full record format and named ‘download.txt’ for compatibility with CiteSpace 6.3. R1, which was then imported into the software. Due to the potential for duplicate records in the database retrieval, the software’s built-in deduplication feature was used to eliminate duplicate entries. This facilitated the analysis of countries, institutions, and keywords, as well as the visualization of cited references and cited authors. During the analysis, issues such as synonymic keywords and inconsistent institution name representations were encountered. To address these, data cleaning was performed first, using Notepad to search for and manually merge synonymic keywords and institution names. The flow chart of the literature screening is shown in Figure 1.

## 3 Result

### 3.1 Analysis of literature

#### 3.1.1 Annual publications

**FIGURE 2:**
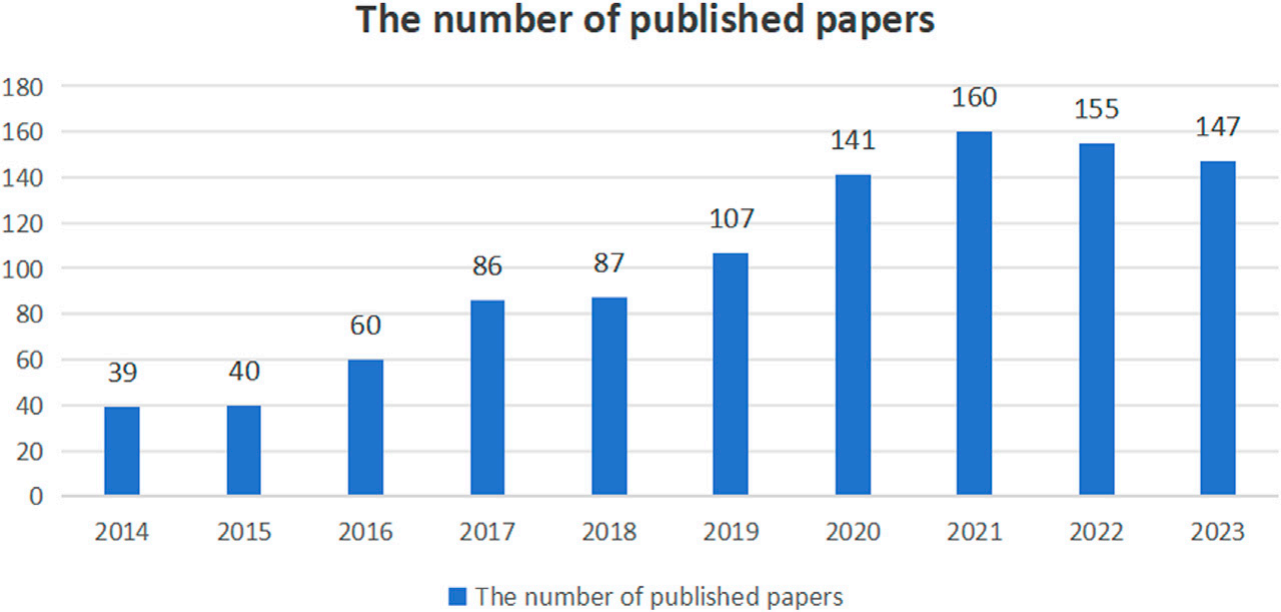
The number of published papers.

In a preliminary search, a total of 1,349 documents published over the past decade on post-stroke exoskeleton-assisted gait training were retrieved. This collection includes articles, conference papers, reviews, and online publications, amounting to 1,336 documents. After removing duplicates, the number of papers was narrowed down to 1,034. As illustrated in Figure 2, the annual publication volume has been increasing year by year, with the highest number of publications recorded in 2021 (160 papers).

#### 3.1.2 Analysis of national (regional) cooperation

**FIGURE 3:**
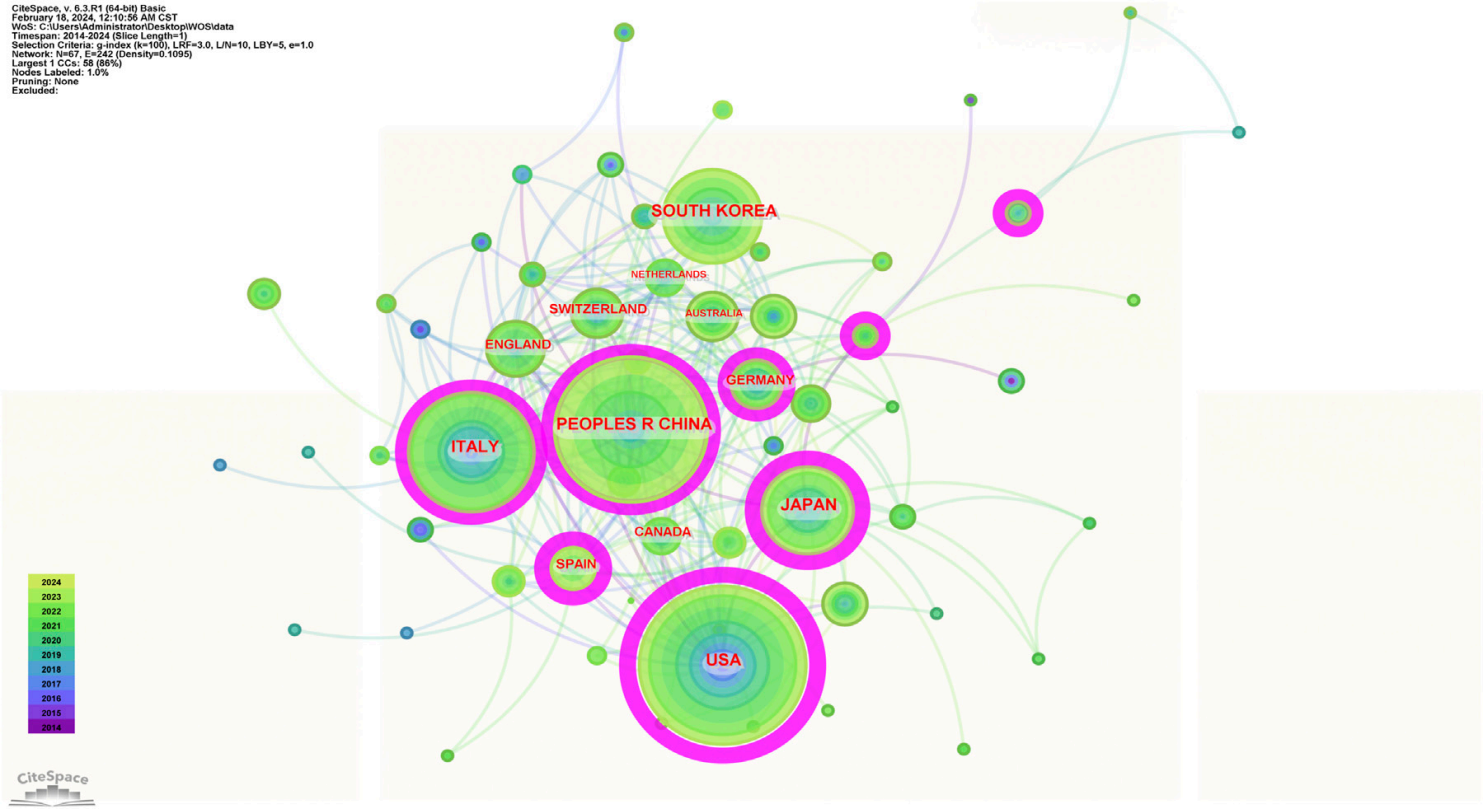
National cooperation analysis map.

In the past decade, a total of 67 countries (regions) have published research related to exoskeleton robot-assisted gait training following stroke, as illustrated in the cooperation map in Figure 3. The cooperation map encompasses 67 nodes and 242 connections, indicating strong collaborative relationships among these countries. The top 5 countries in terms of the number of publications are: the United States (United States of America) with 218 articles, China with 217 articles, Italy with 137 articles, South Korea with 107 articles, and Japan with 92 articles. In terms of betweenness centrality, the top 5 countries are: United States of America with 0.43, Spain with 0.22, Japan with 0.21, Italy with 0.19, and China with 0.15. The United States of America ranks first in both publication volume and betweenness centrality, indicating a leading position among Western countries, while Asian countries such as China, South Korea, and Japan also show strong capabilities in this research area. China ranks second globally in publication volume, but its betweenness centrality is relatively low. A possible reason for this could be China’s recent significant investment in this field, leading to a rapid increase in publications despite a later start.

#### 3.1.3 Institutional collaboration analysis

**FIGURE 4:**
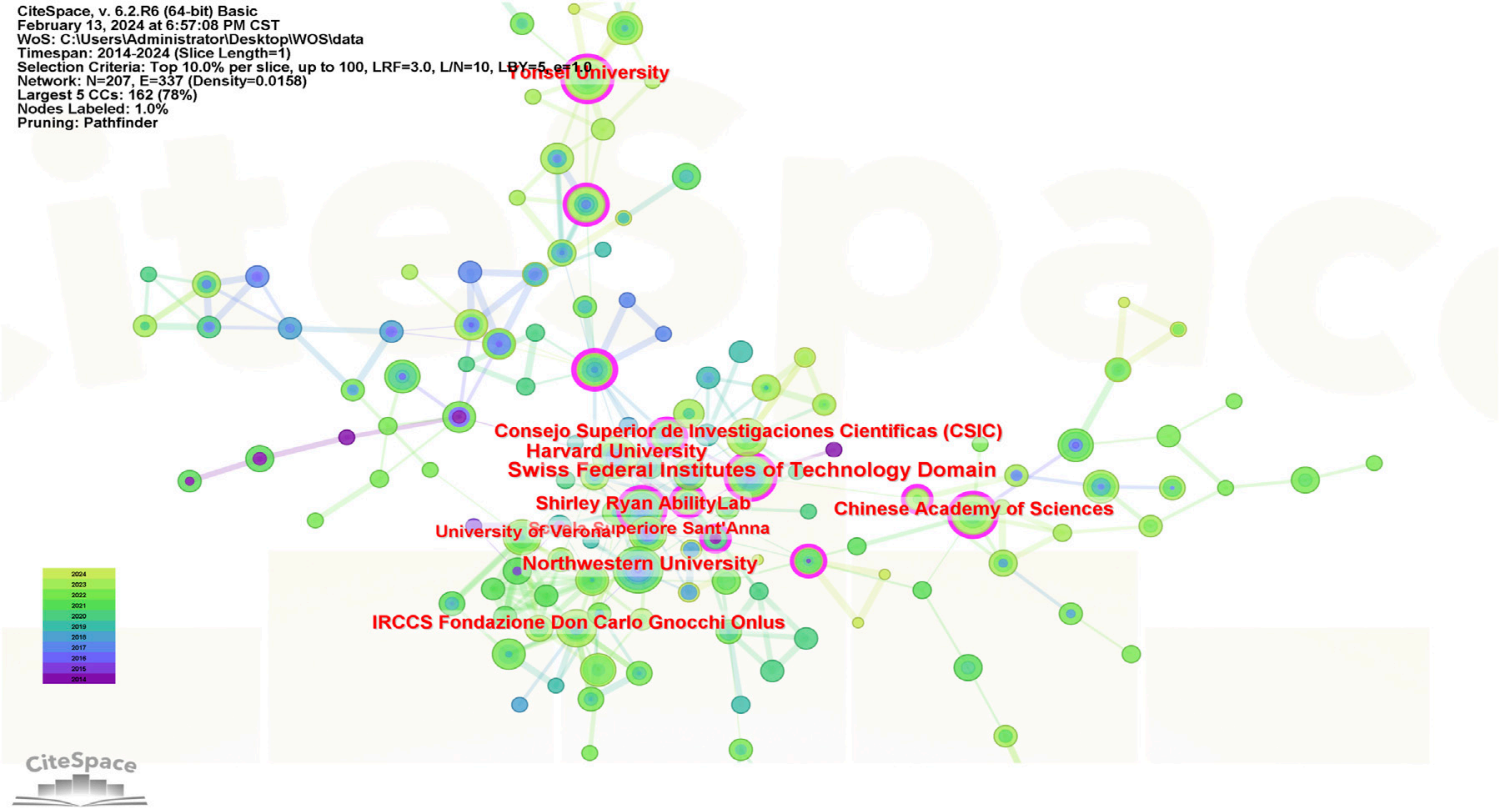
Institutional collaboration analysis map.

In the past decade, a total of 158 institutions have published research related to exoskeleton robot-assisted gait training after stroke, primarily comprising universities from various countries with existing cooperative relationships, as illustrated in the cooperation map shown in Figure 4. The cooperation map features 158 nodes and 268 connections, indicating strong collaborative relationships among these institutions. The top 5 institutions by publication volume are: Swiss Federal Institutes of Technology Domain with 27 articles, Northwestern University (United States of America) with 21 articles, University of Tsukuba (Japan) with 21 articles, Harvard University (United States of America) with 19 articles, and Yonsei University (South Korea) with 18 articles. Regarding betweenness centrality, the top 5 institutions are: Swiss Federal Institutes of Technology Domain (0.19), Shirley Ryan AbilityLab (United States of America) (0.19), Chinese Academy of Sciences (0.13), Yonsei University (South Korea) (0.11), and Harvard University (United States of America) (0.06). Both in terms of publication volume and betweenness centrality, the Swiss Federal Institutes of Technology Domain ranks first, with institutions mainly from Europe and America leading. The Chinese Academy of Sciences ranks third in betweenness centrality.

#### 3.1.4 Co-citation analysis of highly influential authors and cited authors

In the past decade, scholars from around the world have participated in research within this field, with two scholars publishing more than 10 papers each. These are Professor Rocco Salvatore Calabrò from the IRCCS Neurology Center in Italy (18 papers) and Professor Franco Molteni from Valduce Hospital in Italy (10 papers). The top 10 authors in terms of publication volume have relatively low betweenness centrality and citation rates, as shown in Table 1. Over the last 10 years, the top 5 core authors ranked by citation frequency are Mehrholz J from the European Private Scientific Research Institute in Germany (334 citations), Hesse S from the Free University of Berlin in Germany (241 citations), Kwakkel G from the University of Amsterdam in the Netherlands (173 citations), Hornby TG from Northwestern University in the United States of America (152 citations), and MORONE G from the Santa Lucia Foundation in Italy (150 citations). The top 5 core cited authors ranked by betweenness centrality are Mehrholz J from the European Private Scientific Research Institute in Germany (0.13), Banala SK from the University of Delaware in the United States of America (0.09), Hornby TG from Northwestern University in the United States of America (0.08), Schwartz I from Hadassah-Hebrew University in Israel (0.07), and Kawamoto H from the University of Tsukuba in Japan (0.07), as shown in Table 1. Among them, Professor Mehrholz J has the highest citation frequency and betweenness centrality.

**TABLE 1:** Top 10 High-impact Authors and top 10 co-cited Authors in referenced documents.

High-impact author				Co-cited authors of referenced documents			
Author	Country	Number of published papers	Betweenness centrality score	Author	Country	Citation frequency	Betweenness centrality score
Rocco Salvatore Calabro	Italy	18	0.01	Mehrholz J	Germany	334	0.13
Franco Molteni	Italy	10	0	Hesse S	Germany	241	0.06
Yoshiyuki Sankai	Japan	9	0	Kwakkel G	Netherlands	173	0.06
Yasushi Hada	Japan	8	0	Hornby TG	United States of America	152	0.08
Antonino Naro	Italy	8	0	Morone G	Italy	150	0.06
Min Ho Chun	South Korea	8	0	Langhorne P	England	134	0.02
Giovanni Morone	Italy	7	0.01	Hidler J	United States of America	133	0.06
Akira Matsumura	Japan	7	0	Veneman JF	Netherlands	127	0.04
Louis N Awad	United States of America	7	0	Calabrò RS	Italy	126	0.04
Masashi Yamazaki	Japan	7	0	Krebs HI	United States of America	118	0.05

### 3.2 Analysis results of research hotspots

#### 3.2.1 Analysis results of core literature citation and co-citation

**FIGURE 5:**
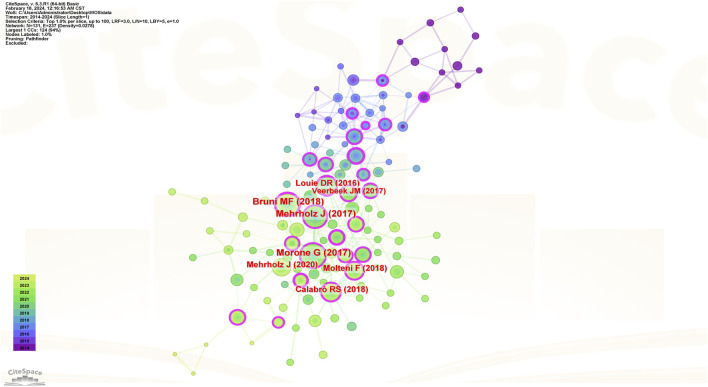
Highly cited core literature analysis map.

Figure 5 reveals that research in this field over the past decade involves a total of 131 core referenced documents. The top five most-cited references are as follows: A document published by Morone G et al. from the Santa Lucia Foundation, Italy, in 2017 on *NEUROPSYCH DIS TREAT*, cited 68 times (Morone et al., 2017); a document by Mehrholz J et al. from the European Private Scientific Research Institute, Germany, in 2017 on *COCHRANE DB SYST REV*, cited 65 times (Mehrholz et al., 2020); a document by Bruni MF et al. from the IRCCS Neurology Center, Italy, in 2018 on *J CLIN NEUROSCI*, cited 61 times (Bruni et al., 2018); and a document by Calabrò RS et al. from the IRCCS Neurology Center, Italy, in 2018 on *J NEUROENG REHABIL*, cited 42 times (Calabrò et al., 2018), and a document by Louie DR et al. from the University of British Columbia, Canada, in 2016 on J *NEUROENG REHABIL*, cited 41 times (Louie and Eng, 2016). Among these five studies, four are reviews, and their findings are similar, all affirming the positive role of robotics in the gait rehabilitation of stroke patients, as detailed in Table 2.

**TABLE 2:** Top 5 cited references of the highest frequency.

Author	Frequency cited	Year	Main content
Morone G (Morone et al., 2017)	68	2017	This paper provides a comprehensive overview of the current advancements and future outlook of robotic technology, encompassing both market-ready systems, in the rehabilitation of post-stroke patients aiming at the restoration of ambulatory functions
Mehrholz J (Mehrholz et al., 2020)	65	2017	The researcher deduced from a meta-analytical approach that post-stroke patients engaging in a regimen of electromechanical-assisted gait training alongside conventional physiotherapy exhibited a higher propensity for regaining autonomous ambulation compared to counterparts abstaining from such technological interventions. This observation was particularly pronounced within the initial 3 months following a cerebrovascular event and was most notable in patients with an initial inability to walk. Future studies are necessitated to ascertain the optimal frequency and length of sessions for electromechanical-assisted gait training to maximize rehabilitative outcomes
Bruni MF (Bruni et al., 2018)	61	2018	The investigator, through a systematic meta-analysis, inferred that the integration of robotic assistance into gait rehabilitation programs could yield favorable results for stroke survivors. Stroke survivors who underwent a combination of robotic device-assisted therapy and conventional physical therapy demonstrated enhanced performance across various measures including the 10-m Walking Test, the 6-Minute Walk Test, the Timed-Up-and-Go, the 5-m Walk Test, and the Functional Ambulation Categories, as compared to those who participated solely in traditional gait training protocols
Calabrò RS (Calabrò et al., 2018)	42	2018	Through a randomized controlled trial, the researcher substantiated that patients with chronic hemiplegia due to stroke exhibited enhanced gait efficacy and neurological plasticity when utilizing the Ekso™ wearable exoskeletons, as opposed to those engaging in conventional terrestrial gait exercises
Louie DR (Louie and Eng, 2016)	41	2016	A scoping review suggested that for individuals with chronic stroke, gait training with exoskeletal support appears to match the efficacy of conventional therapy, whereas those in the sub-acute phase might derive additional advantages from such technologically assisted training

The top five publications ranked by centrality in the intermediary network are as follows: A document by Bruni MF et al. from the IRCCS Neurology Center, Italy, published in *J CLIN NEUROSCI* in 2018, with a centrality score of 0.3 (Bruni et al., 2018); a paper by Louie DR et al. from the University of British Columbia, Canada, in *J NEUROENG REHABIL* in 2016, with a centrality score of 0.28 (Louie and Eng, 2016); a publication by Young AJ et al. from the Georgia Institute of Technology in *IEEE T NEUR SYS REH* in 2017, with a centrality score of 0.25 (Young and Ferris, 2016); a study by Molteni F and team from Valduce Hospital, Italy, in *BRAIN SCI* in 2021, with a centrality score of 0.22 (Molteni et al., 2021); and a document by Morone G et al. from the Santa Lucia Foundation, Italy, in *NEUROPSYCH DIS TREAT* in 2017, with a centrality score of 0.2 (Morone et al., 2017), as detailed in Table 3.

**TABLE 3:** Top 5 cited core references of the betweenness centrality.

Author	Betweenness centrality score	Year	Main content
Bruni MF (Bruni et al., 2018)	0.3	2018	Table 2 shows
Louie DR (Louie and Eng, 2016)	0.28	2016	Table 2 shows
Young AJ (Young and Ferris, 2016)	0.25	2017	This study focused on the actors, sensors, energy sources, materials, and control strategies in the design of lower limb robotic exoskeletons, and discussed the advantages and disadvantages of the emerging technologies and possible futures for the field
Molteni F (Molteni et al., 2021)	0.22	2021	A multicenter randomized controlled trial demonstrated that the clinical efficacy of ground robot assisted gait training (o-RAGT) in subacute stroke patients was similar to that of traditional gait training
Morone G (Morone et al., 2017)	0.2	2017	Table 2 shows

#### 3.2.2 Highly cited literature burst intensity ranking

**FIGURE 6:**
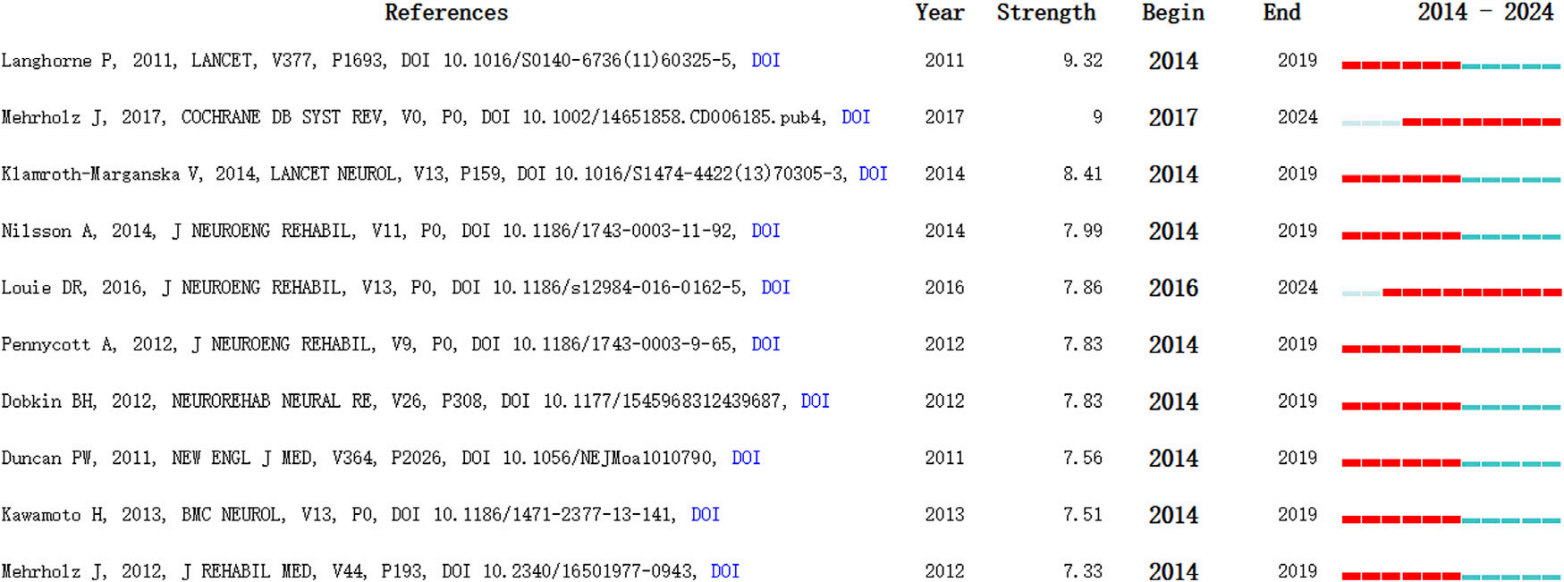
Co-cited references of the burst intensity.

Burst intensity can effectively illustrate the research frontiers within a specific timeframe in a given field, showcasing the areas of concentrated interest and trends. The recent 10-year outlook on the most highly cited publications, ranked by burst intensity in this domain, is presented in Figure 6 (Mehrholz et al., 2020; Louie and Eng, 2016; Duncan et al., 2011; Langhorne et al., 2011; Dobkin and Duncan, 2012; Mehrholz and Pohl, 2012; Pennycott et al., 2012; Kawamoto et al., 2013; Klamroth-Marganska et al., 2014; Nilsson et al., 2014).

#### 3.2.3 Analysis of keyword co-occurrence

**FIGURE 7:**
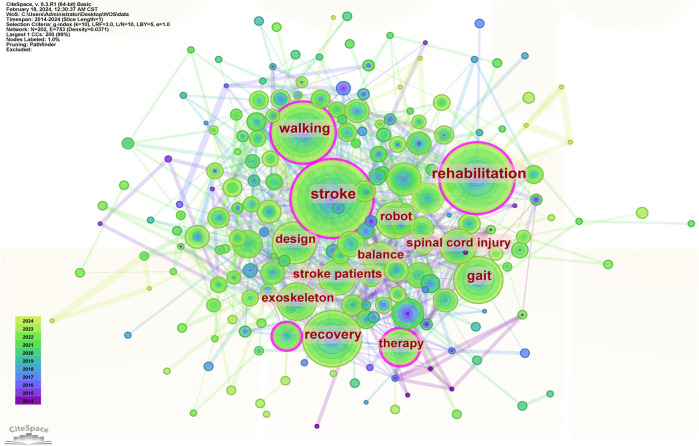
Keyword co-occurrence analysis map.

In the co-occurrence graph of keywords, the size of a node represents the frequency of occurrence of the keyword; the larger the node, the more frequently the keyword appears, indicating a higher popularity of the research topic. The primary metric for measurement is betweenness centrality (ranging from 0 to 1), where a higher value signifies greater influence. A betweenness centrality of ≥0.1 indicates high centrality. As shown in Table 4; Figure 7, the top 10 keywords by betweenness centrality are: rehabilitation, walking, gait rehabilitation, stroke, therapy, body weight support, stroke patients, individuals, upper limb, recovery. The top 10 keywords by frequency of occurrence are: stroke, rehabilitation, walking, recovery, gait, design, spinal cord injury, exoskeleton, therapy, stroke patients.

**TABLE 4:** Top 10 keywords of the betweenness centrality and frequency.

Keyword	Betweenness centrality score	Keyword	Frequency
Rehabilitation	0.17	Stroke	318
Walking	0.15	Rehabilitation	301
Gait rehabilitation	0.14	Walking	237
Stroke	0.11	Recovery	205
Therapy	0.1	Gait	156
Body weight support	0.1	Design	128
Stroke patients	0.09	Spinal cord injury	103
Individuals	0.09	Exoskeleton	98
Upper limb	0.09	Therapy	97
Recovery	0.08	Stroke patients	97

#### 3.2.4 Analysis of keyword co-occurrence clustering

**FIGURE 8:**
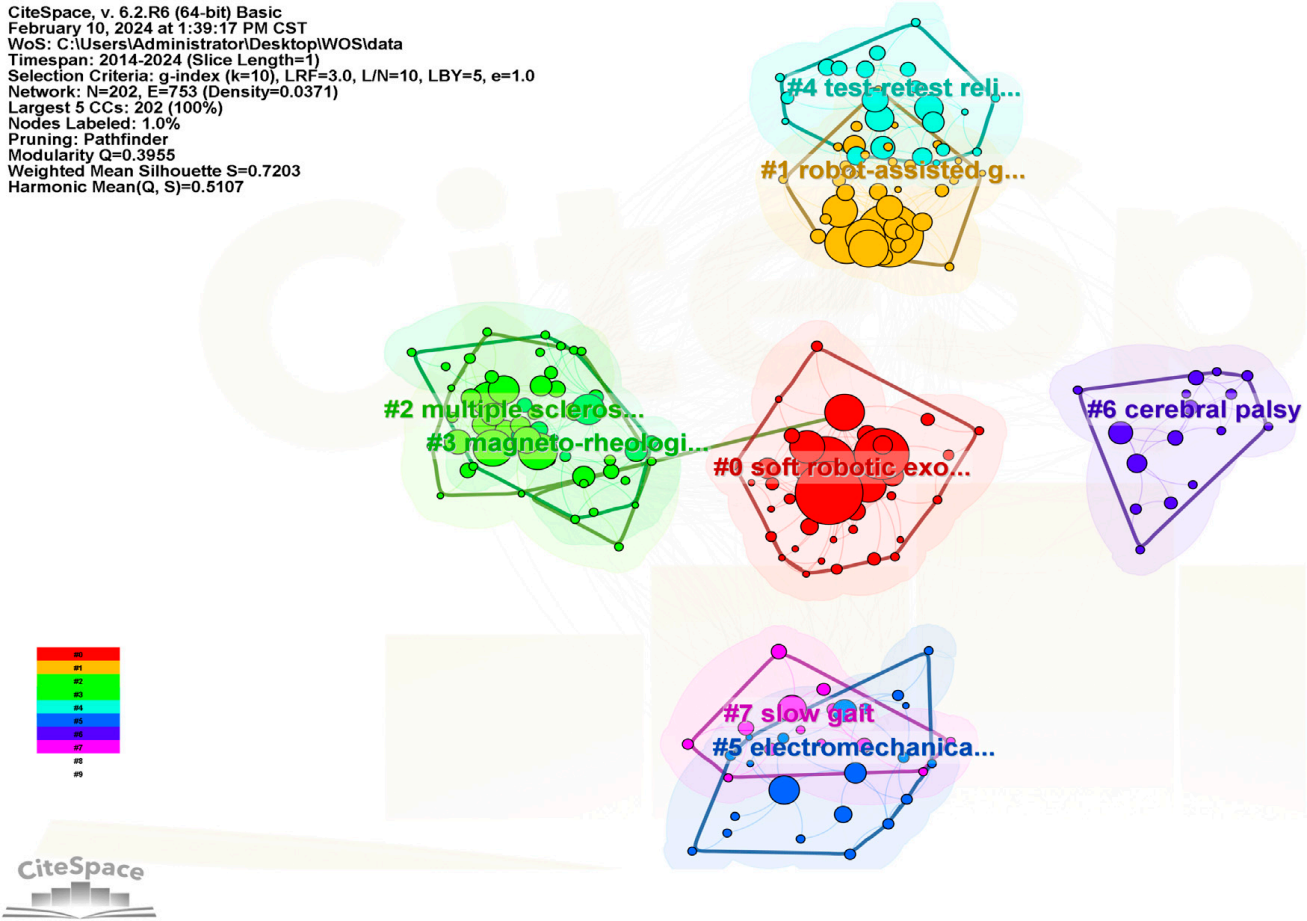
Keyword clustering map.

Keyword co-occurrence clustering analysis is the process of grouping closely related and similar keywords into one category. The greater the number of keywords in a cluster, the smaller the cluster number. The modularity Q represents the clarity of the boundaries between clusters, indicating the significance of the cluster structure. It is generally considered satisfactory if this value is >0.3, indicating clear cluster delineation and significant structure. The Silhouette value measures the degree of closeness between keywords within a cluster, with Silhouette S > 0.5 indicating reasonable clustering. As shown in Figure 8, the cluster modularity Q is 0.3955, and the average silhouette S is 0.7203, suggesting significant cluster structure and high homogeneity. The keywords are divided into 8 clusters: #0 soft robotic exosuit (0.795), #1 robot-assisted gait training (0.717), #2 multiple sclerosis (0.664), #3 magneto-rheological brake (0.619), #4 test-retest reliability (0.673), #5 electromechanical-assisted training (0.77), #6 cerebral palsy (0.754), #7 slow gait (0.891). The keywords of each cluster are listed in Table 5.

**TABLE 5:** Main clusters and contained keywords.

ClusterID	Label (LLR)	Silhouette	Year	Keywords
0	Soft robotic exosuit	0.795	2018	robot-assisted gait training; systematic review; stroke patient; gait training; controlled trial | virtual reality; feasibility study; non-ambulatory patient; robot-assisted gait; using welwalk
1	Robot-assisted gait training	0.717	2017	gait rehabilitation; gait training; systematic review; knee exoskeleton; rehabilitation robot | robot-assisted gait training; chronic stroke patient; pneumatic artificial muscle; robotic rehabilitation; feasibility study
2	Multiple sclerosis	0.664	2016	robot-assisted gait training; chronic stroke patient; gait training; multiple sclerosis; spinal cord injury | systematic review; robotic exoskeleton; chronic stroke; subacute stroke; controlled trial
3	Magneto-rheological brake	0.619	2019	systematic review; robot-assisted gait training; controlled trial; gait training; chronic stroke patient | stroke survivor; randomized controlled trial; robotic therapy; physical human-robot interaction force; case study
4	Test-retest reliability	0.673	2017	motor function; stroke survivor; systematic review; test-retest reliability; randomized controlled trial | robot-mediated rehabilitation; observational feasibility study; italian rehabilitation center; new organizational model; robotic exoskeleton device
5	Electromechanical-assisted training	0.77	2016	robot-assisted gait training; systematic review; stroke patient; gait rehabilitation; effective robot | gait training; chronic stroke; chronic stroke patient; subacute stroke; novel gait training device
6	Cerebral palsy	0.754	2018	systematic review; cerebral palsy; robot-assisted gait training; rehabilitation technologies; advanced robotic therapy individualized gait rehabilitation robotics; before-after study; gait training; hemiplegic patient; same person
7	Slow gait	0.891	2020	robot-assisted gait training; systematic review; stroke patient; intended gait speed prediction; slow gait | randomized controlled trial; overground exoskeleton gait training; inpatient rehabilitation; descriptive analysis; 1-year follow-up study

#### 3.2.5 Analysis of keyword burst and timeline view

**FIGURE 9:**
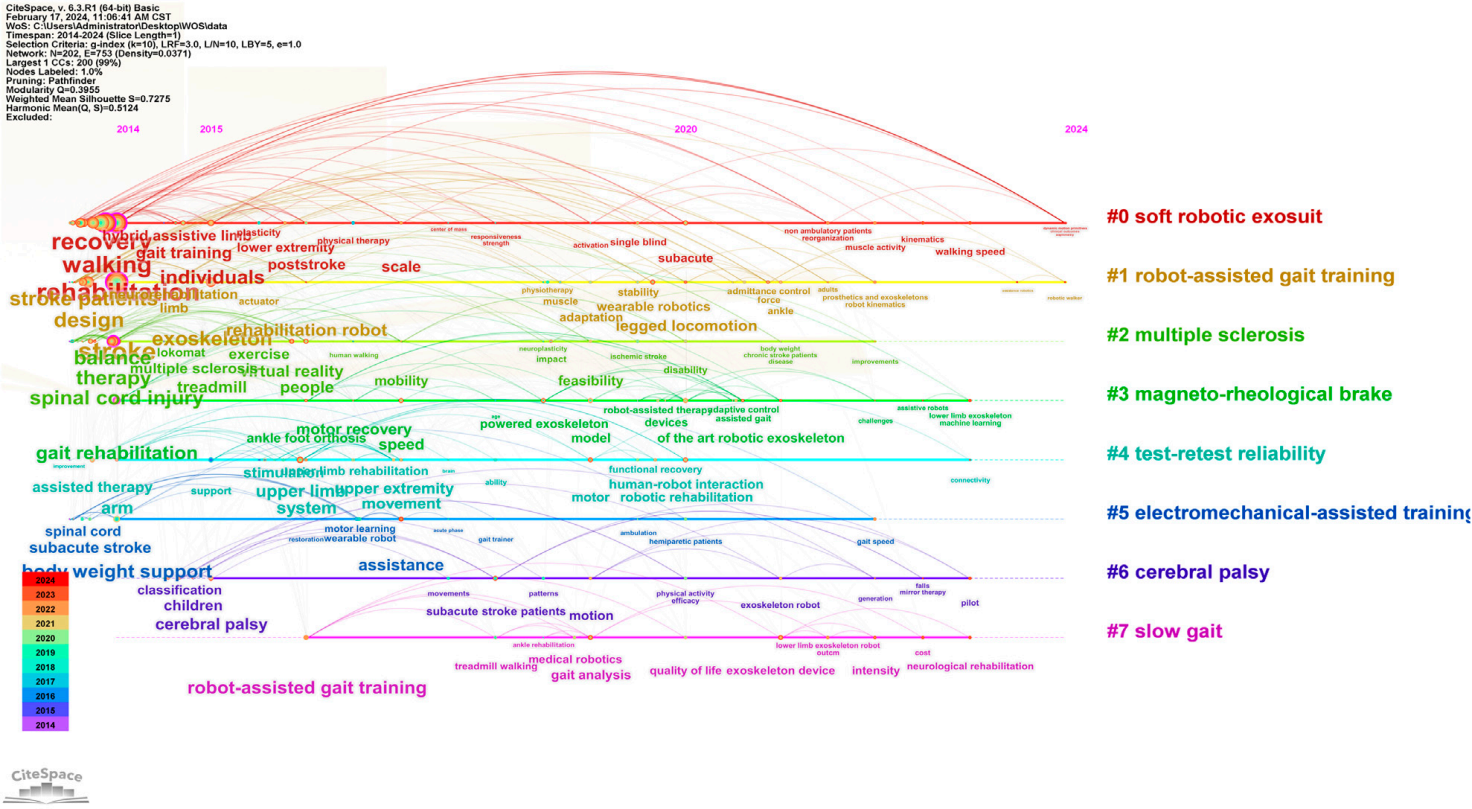
Keyword timeline view.

**FIGURE 10:**
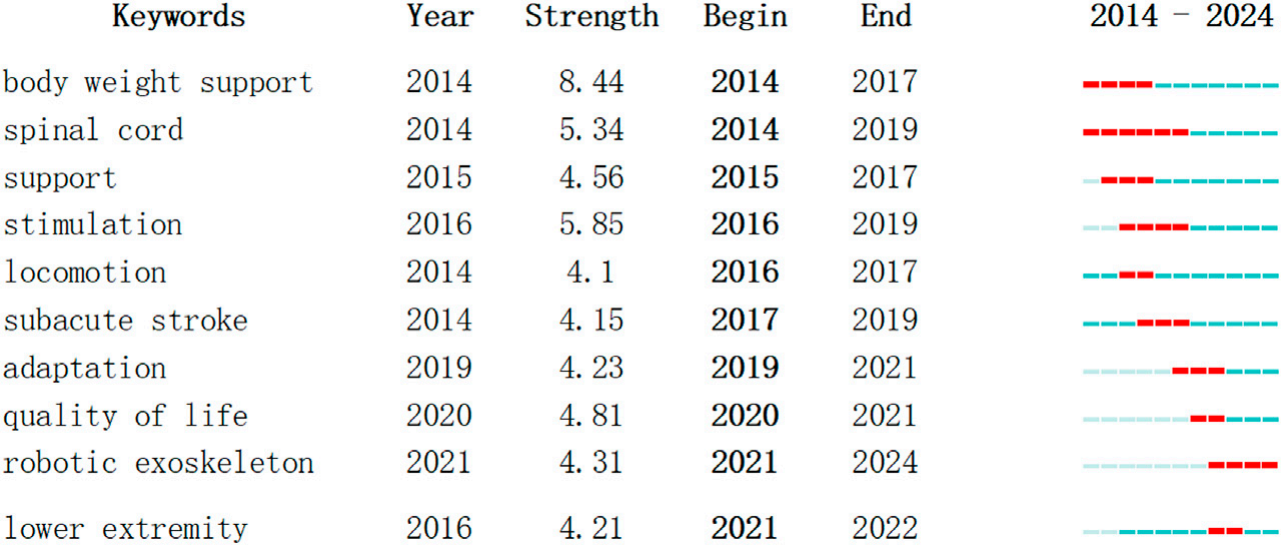
Ranking of keywords by burst strength.

Keyword burst refers to the sudden appearance of keywords within a certain period of time, representing the development trend of research. It can effectively showcase the different research frontiers at various time stages, presenting the hotspots and trends of the field. ‘Year’ indicates the first year the keyword appeared, ‘Begin’ and ‘End’ represent the start and end time of the keyword’s occurrence, respectively. ‘Strength’ denotes the intensity of burst, where a higher value indicates greater burst strength. It is generally considered that only when a keyword’s burst strength exceeds 3 can it be termed an emerging keyword. A timeline view, created after generating the co-citation cluster map, uses cluster numbers as the *Y*-axis and the publication years of citations as the *X*-axis to draw a knowledge map. As shown in Figures 9, 10, the burst of keywords in this research field can be roughly divided into two periods: (1) From 2014 to 2020, the research focus was on exploring the effectiveness and reliability of RE in assisting stroke patients with gait training. The movement patterns of gait training were continuously adjusted in practice, and the types and functions of RE were constantly optimized and iterated. (2) From 2021 to 2024, the emerging hotspots shifted to quality of life, machine learning, and cost.

## 4 Discussion

### 4.1 Distribution of countries, institutions, authors, and co-citation analysis

In terms of publication volume and betweenness centrality, the United States of America ranks first, overall indicating that Western countries lead in this field, while Asian countries such as China, South Korea, and Japan also possess strong capabilities. This may relate to the economic strength of these countries, policy directions, and research funding orientations. The United States has the highest number of publications globally and has always been at the forefront of technological innovation and artificial intelligence. As a populous country, China has seen an increasing incidence and number of stroke patients (Wu et al., 2019), thus prioritizing related research. Being a manufacturing powerhouse, China has rapidly developed in the field of artificial intelligence in recent years, leading to swift advancements and applications of RE. Notably, although China ranks second globally in publication volume, its betweenness centrality is relatively low. This may be due to the country’s significant investment in this field in recent years, leading to a rapid increase in publications. However, due to a later start in technology, its performance in academic collaboration appears less favorable. While Switzerland does not dominate in publication volume, the Swiss Federal Institutes of Technology Domain has shown exceptional performance in institutional cooperation, possibly due to its early start in research and technical accumulation in engineering and technology fields.

Overall, authors with a high frequency of citations and betweenness centrality in the cited literature are predominantly scholars in the medical field, with few from engineering disciplines. This might be attributed to the fact that although the development of RE poses a technological challenge, the clinical efficacy of such devices requires long-term and careful observation. Hence, scholars in the medical field tend to have their publications cited more frequently in this area. Among them, the most frequently cited and highest betweenness centrality author is Professor J. Mehrholz from the European Private Scientific Research Institute and the Carl Gustav Carus Faculty of Medicine at Dresden University of Technology in Germany. Professor Mehrholz’s research primarily focuses on functional impairments caused by conditions such as stroke, Parkinson’s disease, and myasthenia, and is dedicated to evaluating the effectiveness of common rehabilitation methods in the neurology field through evidence-based medicine. His research direction in the field of motor rehabilitation for stroke patients includes electromechanical-assisted training, robotic-assisted therapy, and other means to improve lower limb function (Hesse et al., 2008; Mehrholz and Pohl, 2012; Mehrholz, 2016; Mehrholz et al., 2017; Wu et al., 2019).

Highly cited papers often include reviews and systematic reviews, likely because research related to RE mainly consists of comparative studies. However, due to constraints related to research subjects, intervention measures, ethics, etc., initial studies tend to have smaller sample sizes (Leow et al., 2023). Meta-analyses increase the sample size by pooling study results, thereby offering a more holistic and comprehensive analysis of intervention effects, leading to a higher citation count. However, due to inherent limitations of meta-analyses, such as issues with heterogeneity, publication bias, and significant influence from the quality of original studies (Khan et al., 1996; Hatala et al., 2005)^,^ meta-analyses cannot replace original research. The field requires more large-scale, multicenter randomized controlled trials to provide high-quality evidence in the future.

### 4.2 The research status and hotspots of RE assisting in gait therapy for stroke patients

From the standpoint of publication volume and timing, the application of RE in facilitating gait rehabilitation for patients with stroke-induced hemiparesis has attracted increasing scholarly interest since the early 21st century, with a significantly growing body of literature observed. Notably, a marked increase in publications has been evident since 2016. This trend can likely be attributed to the advanced maturation of RE technology and its evolution from a phase of technological exploration to broader market and application phases (Calafiore et al., 2022). According to an analysis of keyword co-occurrence and clustering, the past decade’s prominent research topics have encompassed the technology of RE, including both hardware and control strategies, alongside their clinical applications. Prior to 2016, technological research predominantly focused on body weight support and motion control within RE systems. Following 2020, machine learning has become a pivotal area of research interest. In the domain of RE applications, the period leading up to 2020 saw intense research focus on evaluating the efficacy and safety outcomes. After 2020, the academic community has increasingly turned its attention towards examining the impact of RE on quality of life and considerations of cost analysis.

#### 4.2.1 RE hardware devices

Currently, there are two types of RE: rigid exoskeletons and soft exoskeletons. Accroding to keyword co-occurrence clustering analysis, “soft exoskeletons” was the hotspots. In the analysis of keyword co-occurrence, the high frequency of occurrence for “weight support” indicates that the weight ratio (the self-weight of the exoskeleton and its maximum load capacity) is a technical research focal point in the hardware configuration of exoskeletons. The average weight of exoskeletons used for stroke patients is 8.90 ± 7.48 kg, with a maximum carrying weight of 100 kg (Rodríguez-Fernández et al., 2021). The more joint drives an RE has, the heavier it becomes, and the overweight exoskeleton becomes a problem for patients to wear and carry (Tefertiller et al., 2018). Soft exoskeletons, due to their different materials, tend to be lighter than rigid exoskeletons (Sanchez-Villamañan et al., 2019), but their load-bearing capacity is less than that of rigid exoskeletons, therefore their use is limited in patients with severe muscle weakness and may increase the risk of falling (Awad et al., 2017). Stroke patients have a higher rate of obesity (Ekker et al., 2018), and although the weight of stroke survivors decreases post-stroke, during the subacute phase of stroke—a critical period for recovery—the patient’s weight does not suddenly drop. Therefore, when designing, a broader audience needs to be considered, and engineers need to consider reducing the self-weight of the exoskeleton while increasing its load-bearing capacity.

#### 4.2.2 Control strategies of RE

Scholars such as De Miguel-Fernández, J, in their work (de Miguel-Fernández et al., 2023), focus on the research progress in incorporating fall detection, balance recovery, and stability assurance strategies in the design and application of lower limb exoskeletons. They provide an overview of the current control strategy framework for lower limb exoskeletons and analyze the advantages and disadvantages of common methods used for stable walking with exoskeletons (including zero moment point, center of mass, and extrapolated center of mass). The study finds that the current level of evidence regarding the effectiveness of different machine control strategies on clinical outcomes for patients is not high. This is mainly due to the limited number of studies on RE with different control strategies within the same series and the lack of uniformity in research subjects and clinical outcome indicators, leading to high heterogeneity in clinical results. It is suggested that future research should focus more on standardized comparisons between control strategies, analyze the relationship between control parameters and biomechanical indicators, and reduce biases caused by hardware heterogeneity and patients’ own recovery and compensation strategies. David Pinto-Fernandez et al. (Pinto-Fernandez et al., 2020) note that the number of papers assessing robot-assisted motion is growing exponentially, with almost half of the papers focusing on walking on level ground or treadmills. This highlights that the current research hotspot on exoskeleton motion control strategies is still on achieving basic motor skills. However, the study of control strategies should go beyond the achievement of basic motor skills to more extensively focus on how these strategies can be effectively translated into clinical practice, particularly in complex and unpredictable outdoor environments. In the clinical setting, the control strategies for RE require further refinement and personalization to cater to the specific needs and rehabilitation stages of different patients (Tian et al., 2024). As demonstrated in the framework proposed by Hohl et al. (Hohl et al., 2022) clinicians should consider the patient’s specific impairments, device characteristics, and the overall goals of the treatment plan when selecting and implementing exoskeleton technology. This includes a detailed matching of the support level provided by the device, assistance/resistance modes, feedback enhancement, and the minimum functionality required.

#### 4.2.3 The efficacy of RE-assisted walking therapy for stroke patients

The effectiveness of RE in stroke patients primarily pertains to the correction of abnormal gait patterns and the enhancement of walking ability. Additional reported benefits include improved cardiopulmonary function, alleviation of lower limb spasticity, enhancement of patients’ balance capabilities, promotion of proprioceptive recovery, and increased neural plasticity (Molteni et al., 2020; Herrin et al., 2023; Lee et al., 2023; Rey-Prieto et al., 2023). Currently, most RE on the market offer dual functionalities, as lower limb rehabilitation in clinical settings is predominantly based on gait. Initiating walking training too early in acute stroke patients can lead to abnormal movement patterns due to insufficient leg strength and balance (Plummer et al., 2007). RE can simulate normal gait patterns in a weight-reduced state and adjust the level of assistance based on the patient’s gait changes, thus helping patients establish a normal gait while also improving muscle strength and walking distance. Commonly used criteria for assessing the promotion of lower limb rehabilitation by RE include walking ability tests, muscle tone and strength assessments, joint range of motion measurements, balance ability assessments, gait analysis (including kinematic and kinetic analyses, and dynamic electromyography tracking), and surface electromyography. Calabrò et al. conducted a clinical trial on neuroplasticity in stroke patients utilizing powered exoskeletons, finding that the exoskeletons also impact the wearer’s neuromuscular control, facilitating the restoration of specific cerebral plasticity mechanisms. Although RE are generally beneficial for lower limb rehabilitation and social reintegration in hemiplegic stroke patients, the current evidence is insufficient for clinical physicians to select specific treatment plans due to variations in RE types, treatment dosages, stages of stroke patients, and disease severity. This has become one of the barriers to the clinical application of RE. Future research could consider comparing different RE, treatment doses, and stroke populations to provide more detailed and reliable treatment strategies for clinical physicians, guiding the selection and use of RE for patients with different clinical needs.

The effectiveness of RE is of paramount concern for developers and clinical researchers, yet it is important to note that research on user experience is relatively scarce (Louie et al., 2020; Morone et al., 2017). Given that the end users and operators of exoskeletons are patients and professional healthcare personnel, it is essential to thoroughly understand the experiences of these two groups when utilizing RE. Such insights are vital for directing the future development and optimization of RE technology. Researchers like Julie Vaughan-Graham have explored the experiences of stroke patients and physical therapists with the Exo-H2 exoskeleton (a powered exoskeleton) in gait rehabilitation. Physical therapists believe that improvements are necessary in the efficiency of donning and doffing the technology, the convenience of its operation, the diversity of parameter settings, and the real-time output of data. Concurrently, they express concerns regarding the balance of the RE and the potential musculoskeletal damage they may cause. Patients have an optimistic view of the technology, but because the exoskeleton’s pre-programmed modes control their movements, it makes them feel as though the exoskeleton is walking for them, rather than assisting. They prefer the exoskeleton to aid in walking, not to replace it. Thus, enhancing the interaction between RE and users, as well as the self-balancing features of exoskeleton are likely challenges that technicians will need to address in the future (Vaughan-Graham et al., 2020).

### 4.3 Research trends in RE-assisted walking therapy for stroke patients

In the field of RE technology research, RE have evolved from strict gait standardization control towards a shared control with human-machine interaction. While standardization control can correct a patient’s gait to match the trajectory of a healthy person’s gait, achieving a seemingly corrective objective, it also limits active engagement with the machine, potentially reducing the patient’s motivation (Wu, 2021). Shared control addresses this issue more effectively, currently, shared control technology mainly utilizes theories such as adaptive fuzzy control, admittance control, impedance control, and gait trajectory planning as a basis to adjust and optimize leg movements during a patient’s walking process (Rajasekaran et al., 2018). However, the application of current technology still seems to fall short of idealized interaction, with patients not being entirely satisfied with the experience. Future research hotspots and trends include exoskeleton human-machine symbiotic design, motion intention detection, human-machine hybrid system motion control, and the integration of technologies such as virtual reality and machine learning (Postol et al., 2021).

Generally, RE exert a positive effects on therapeutic effectiveness and the enhancement of quality of life. However, it is worth noting that current application research is primarily focused on patients in the subacute phase of stroke, which may be attributed to the critical importance of correcting gait and restoring walking ability during this period. Appropriate interventions during this phase can help reduce sequelae and improve long-term outcomes (Calafiore et al., 2022). However, with the decline in mortality rates among stroke patients (Benjamin et al., 2019), the life expectancy of patients in the chronic phase has extended. The capacity for mobility and self-care within this demographic necessitates proactive intervention and enhancement. Consequently, the efficacy of applications amongst such populations must also be duly reported. Additionally, previous studies often selected hospitals or specific rehabilitation treatment site due to easier access to patients and the presence of professional medical staff to prevent injuries. However, with the maturation and expansion of RE technology, future applications in homes and communities might become a trend, considering that most stroke survivors spend a significant amount of their time at home or in the community, rather than in rehabilitation institutions or hospitals.

## 5 Limitation

In terms of data, this article only retrieved documents from the Web of Science Core Collection, omitting searches in other databases such as PUBMED, Embase, and related databases. This exclusion may lead to the omission of some relevant articles in the field. Secondly, the types of documents included in this study were primarily English-language papers and reviews, thereby potentially overlooking the contributions of high-quality works published in other languages or formats. Finally, since the data of this study, such as citation counts and co-occurrence frequencies, are related to the publication date, it is possible that some high-quality articles that were published more recently may have been overlooked.

## 6 Conclusion

This study synthesizes a decade of research on robotic exoskeletons for stroke rehabilitation, revealing a surge in publications and technological evolution. The United States, China, and several European countries lead with significant contributions. Initial focus on exoskeleton development has transitioned to integrating AI and optimizing user interaction. High-impact reviews consolidate evidence, yet there’s a call for more rigorous trials. Technological strides from rigid to soft exoskeletons address weight and usability, with control strategies advancing for patient comfort and safety. Looking forward, the field is set to focus on human-machine symbiosis, motion intention detection, and the integration of virtual reality and machine learning, with potential for home and community-based rehabilitation. Future research should prioritize investigations within the cohort of chronic stroke patients, while concurrently attending to the experiential accounts of both patients and rehabilitation therapists.

## References

[B1] Awad L. N. Bae J. O’donnell K. De Rossi S. M. Hendron K. Sloot L. H. (2017). A soft robotic exosuit improves walking in patients after stroke. Sci. Transl. Med. 9 (400), eaai9084. 10.1126/scitranslmed.aai9084 28747517

[B2] Benjamin E. J. Muntner P. Alonso A. Bittencourt M. S. Callaway C. W. Carson A. P. (2019). Heart disease and stroke statistics—2019 update: a report from the American Heart Association. Circulation 139 (10), e56–e528. 10.1161/cir.0000000000000659 30700139

[B3] Bruni M. F. Melegari C. De Cola M. C. Bramanti A. Bramanti P. Calabrò R. S. (2018). What does best evidence tell us about robotic gait rehabilitation in stroke patients: a systematic review and meta-analysis. J. Clin. Neurosci. 48, 11–17. 10.1016/j.jocn.2017.10.048 29208476

[B4] Calabrò R. S. Naro A. Russo M. Bramanti P. Carioti L. Balletta T. (2018). Sha** neuroplasticity by using powered exoskeletons in patients with stroke: a randomized clinical trial. J. neuroengineering rehabilitation 15 (1), 1–16. 10.1186/s12984-018-0377-8 PMC5918557 29695280

[B6] Calafiore D. Negrini F. Tottoli N. Ferraro F. Ozyemisci-Taskiran O. de Sire A. (2022). Efficacy of robotic exoskeleton for gait rehabilitation in patients with subacute stroke: a systematic review. Eur. J. Phys. Rehabilitation Med. 58 (1), 1–8. 10.23736/s1973-9087.21.06846-5 PMC9980569 34247470

[B7] Cheng C. J. Yu H. B. (2024). Global trends and development of acupuncture for stroke: a review and bibliometric analysis. Medicine 103 (3), e36984. 10.1097/md.0000000000036984 38241541 PMC10798747

[B8] Chi X. Fan X. Fu G. Liu Y. Zhang Y. Shen W. (2023). Research trends and hotspots of post-stroke cognitive impairment: a bibliometric analysis. Front. Pharmacol. 14, 1184830. 10.3389/fphar.2023.1184830 37324494 PMC10267734

[B9] de Miguel-Fernández J. Lobo-Prat J. Prinsen E. Font-Llagunes J. M. Marchal-Crespo L. (2023). Control strategies used in lower limb exoskeletons for gait rehabilitation after brain injury: a systematic review and analysis of clinical effectiveness. J. neuroengineering rehabilitation 20 (1), 23. 10.1186/s12984-023-01144-5 PMC9938998 36805777

[B53] de Miguel Fernandez J. Rey-Prieto M. Rio M. S. Lopez-Matas H. Guirao-Cano L. Font-Llagunes J. M. Lobo-Prat J. (2023). Adapted Assistance and Resistance Training With a Knee Exoskeleton After Stroke. IEEE transactions on neural systems and rehabilitation engineering: a publication of the IEEE Engineering in Medicine and Biology Society, 31, 3265–3274. 10.1109/TNSRE.2023.3303777 37556332

[B10] Dobkin B. H. Duncan P. W. (2012). Should body weight–supported treadmill training and robotic-assistive steppers for locomotor training trot back to the starting gate? Neurorehabilitation neural repair 26 (4), 308–317. 10.1177/1545968312439687 22412172 PMC4099044

[B11] Dong Y. Weng L. Hu Y. Mao Y. Zhang Y. Lu Z. (2022). Exercise for stroke rehabilitation: a bibliometric analysis of global research from 2001 to 2021. Front. Aging Neurosci. 14, 876954. 10.3389/fnagi.2022.876954 35783146 PMC9247282

[B12] Donthu N. Kumar S. Mukherjee D. Pandey N. Lim W. M. (2021). How to conduct a bibliometric analysis: an overview and guidelines. J. Bus. Res. 133, 285–296. 10.1016/j.jbusres.2021.04.070

[B13] Duncan P. W. Sullivan K. J. Behrman A. L. Azen S. P. Wu S. S. Nadeau S. E. (2011). Body-weight–supported treadmill rehabilitation after stroke. N. Engl. J. Med. 364 (21), 2026–2036. 10.1056/nejmoa1010790 21612471 PMC3175688

[B14] Ekker M. S. Boot E. M. Singhal A. B. Tan K. S. Debette S. Tuladhar A. M. (2018). Epidemiology, aetiology, and management of ischaemic stroke in young adults. Lancet Neurology 17 (9), 790–801. 10.1016/s1474-4422(18)30233-3 30129475

[B15] Feigin V. L. Brainin M. Norrving B. Martins S. Sacco R. L. Hacke W. (2022). World Stroke Organization (WSO): global stroke fact sheet 2022. Int. J. Stroke 17 (1), 18–29. 10.1177/17474930211065917 34986727

[B16] Feigin V. L. Stark B. A. Johnson C. O. Roth G. A. Bisignano C. Abady G. G. (2021). Global, regional, and national burden of stroke and its risk factors, 1990–2019: a systematic analysis for the Global Burden of Disease Study 2019. Lancet Neurology 20 (10), 795–820. 10.1016/s1474-4422(21)00252-0 34487721 PMC8443449

[B17] Harjpal P. Kovela R. K. Jain M. Kovela Sr R. K. (2022). Bilateral lower limb training for post-stroke survivors: a bibliometric analysis. Cureus 14 (9), e29615. 10.7759/cureus.29615 36321041 PMC9603067

[B18] Hatala R. Keitz S. Wyer P. Guyatt G. (2005). Tips for learners of evidence-based medicine: 4. Assessing heterogeneity of primary studies in systematic reviews and whether to combine their results. Cmaj 172 (5), 661–665. 10.1503/cmaj.1031920 15738493 PMC550638

[B19] Herrin K. Upton E. Young A. (2023). Towards meaningful community ambulation in individuals post stroke through use of a smart hip exoskeleton: a preliminary investigation. Assist. Technol., 1–11. 10.1080/10400435.2023.2239555 37493447

[B20] Hesse S. Mehrholz J. Werner C. (2008). Robot-assisted upper and lower limb rehabilitation after stroke: walking and arm/hand function. Dtsch. Ärzteblatt Int. 105 (18), 330–336. 10.3238/arztebl.2008.0330 PMC2707632 19629252

[B21] Hohl K. Giffhorn M. Jackson S. Jayaraman A. (2022). A framework for clinical utilization of robotic exoskeletons in rehabilitation. J. neuroengineering rehabilitation 19 (1), 115. 10.1186/s12984-022-01083-7 PMC9618174 36309686

[B22] Hu Y. Yu Z. Cheng X. Luo Y. Wen C. (2020). A bibliometric analysis and visualization of medical data mining research. Medicine 99 (22), e20338. 10.1097/md.0000000000020338 32481411 PMC7748217

[B23] Kawamoto H. Kamibayashi K. Nakata Y. Yamawaki K. Ariyasu R. Sankai Y. (2013). Pilot study of locomotion improvement using hybrid assistive limb in chronic stroke patients. BMC Neurol. 13, 141–148. 10.1186/1471-2377-13-141 24099524 PMC3851710

[B24] Khan K. S. Daya S. Jadad A. R. (1996). The importance of quality of primary studies in producing unbiased systematic reviews. Archives Intern. Med. 156 (6), 661–666. 10.1001/archinte.156.6.661 8629879

[B25] Kim C. Kim H. J. (2022). Effect of robot-assisted wearable exoskeleton on gait speed of post-stroke patients: a systematic review and meta-analysis of a randomized controlled trials. Phys. Ther. Rehabilitation Sci. 11 (4), 471–477. 10.14474/ptrs.2022.11.4.471

[B26] Klamroth-Marganska V. Blanco J. Campen K. Curt A. Dietz V. Ettlin T. (2014). Three-dimensional, task-specific robot therapy of the arm after stroke: a multicentre, parallel-group randomised trial. Lancet Neurology 13 (2), 159–166. 10.1016/s1474-4422(13)70305-3 24382580

[B27] Koenig A. Omlin X. Bergmann J. Zimmerli L. Bolliger M. Müller F. (2011). Controlling patient participation during robot-assisted gait training. J. neuroengineering rehabilitation 8, 14–12. 10.1186/1743-0003-8-14 PMC3076234 21429200

[B28] Krishnamurthi R. V. Ikeda T. Feigin V. L. (2020). Global, regional and country-specific burden of ischaemic stroke, intracerebral haemorrhage and subarachnoid haemorrhage: a systematic analysis of the global burden of disease study 2017. Neuroepidemiology 54 (2), 171–179. 10.1159/000506396 32079017

[B29] Langhorne P. Bernhardt J. Kwakkel G. (2011). Stroke rehabilitation. Lancet 377 (9778), 1693–1702. 10.1016/s0140-6736(11)60325-5 21571152

[B30] Lee Y. H. Ko L. W. Hsu C. Y. Cheng Y. Y. (2023). Therapeutic effects of robotic-exoskeleton-assisted gait rehabilitation and predictive factors of significant improvements in stroke patients: a randomized controlled trial. Bioengineering 10 (5), 585. 10.3390/bioengineering10050585 37237654 PMC10215135

[B31] Leow X. R. G. Ng S. L. A. Lau Y. (2023). Overground robotic exoskeleton training for patients with stroke on walking-related outcomes: a systematic review and meta-analysis of randomized controlled trials. Archives Phys. Med. Rehabilitation 104, 1698–1710. 10.1016/j.apmr.2023.03.006 36972746

[B32] Li C. Shu X. Liu X. (2022). Research hotspots and frontiers in post stroke pain: a bibliometric analysis study. Front. Mol. Neurosci. 15, 905679. 10.3389/fnmol.2022.905679 35645732 PMC9137410

[B33] Loro A. Borg M. B. Battaglia M. Amico A. P. Antenucci R. Benanti P. (2023). Balance rehabilitation through robot-assisted gait training in post-stroke patients: a systematic review and meta-analysis. Brain Sci. 13 (1), 92. 10.3390/brainsci13010092 36672074 PMC9856764

[B34] Louie D. R. Eng J. J. (2016). Powered robotic exoskeletons in post-stroke rehabilitation of gait: a sco** review. J. neuroengineering rehabilitation 13, 1–10. 10.1186/s12984-016-0162-5 PMC4898381 27278136

[B35] Louie D. R. Mortenson W. B. Durocher M. Teasell R. Yao J. Eng J. J. (2020). Exoskeleton for post-stroke recovery of ambulation (ExStRA): study protocol for a mixed-methods study investigating the efficacy and acceptance of an exoskeleton-based physical therapy program during stroke inpatient rehabilitation. BMC Neurol. 20 (1), 35–39. 10.1186/s12883-020-1617-7 31992219 PMC6988257

[B36] Mehrholz J. (2016). Towards evidence-based practice of technology-based gait rehabilitation after stroke. Physiother. Res. Int. J. Res. Clin. Phys. Ther. 21 (4), 201–202. 10.1002/pri.1680 27860155

[B37] Mehrholz J. Pohl M. (2012). Electromechanical-assisted gait training after stroke: a systematic review comparing end-effector and exoskeleton devices. J. rehabilitation Med. 44 (3), 193–199. 10.2340/16501977-0943 22378603

[B38] Mehrholz J. Thomas S. Elsner B. (2017). Treadmill training and body weight support for walking after stroke. Cochrane database Syst. Rev. 2017 (8), CD002840. 10.1002/14651858.cd002840.pub4 PMC6483714 28815562

[B39] Mehrholz J. Thomas S. Kugler J. Pohl M. Elsner B. (2020). Electromechanical‐assisted training for walking after stroke. Cochrane database Syst. Rev. 2020 (10), CD006185. 10.1002/14651858.cd006185.pub5 PMC8189995 33091160

[B40] Molteni F. Formaggio E. Bosco A. Guanziroli E. Piccione F. Masiero S. (2020). Brain connectivity modulation after exoskeleton-assisted gait in chronic hemiplegic stroke survivors: a pilot study. Am. J. Phys. Med. rehabilitation 99 (8), 694–700. 10.1097/phm.0000000000001395 32084035

[B41] Molteni F. Guanziroli E. Goffredo M. Calabrò R. S. Pournajaf S. Gaffuri M. (2021). Gait recovery with an overground powered exoskeleton: a randomized controlled trial on subacute stroke subjects. Brain Sci. 11 (1), 104. 10.3390/brainsci11010104 33466749 PMC7830339

[B42] Morone G. Paolucci S. Cherubini A. De Angelis D. Venturiero V. Coiro P. (2017). Robot-assisted gait training for stroke patients: current state of the art and perspectives of robotics. Neuropsychiatric Dis. Treat. Vol. 13, 1303–1311. 10.2147/ndt.s114102 PMC5440028 28553117

[B43] Nilsson A. Vreede K. S. Häglund V. Kawamoto H. Sankai Y. Borg J. (2014). Gait training early after stroke with a new exoskeleton–the hybrid assistive limb: a study of safety and feasibility. J. neuroengineering rehabilitation 11, 92–11. 10.1186/1743-0003-11-92 PMC4065313 24890413

[B44] Ninkov A. Frank J. R. Maggio L. A. (2022). Bibliometrics: methods for studying academic publishing. Perspect. Med. Educ. 11 (3), 173–176. 10.1007/s40037-021-00695-4 34914027 PMC9240160

[B45] Ogihara H. Yamamoto N. Kurasawa Y. Kamo T. Hagiyama A. Hayashi S. (2023). Characteristics and methodological quality of the top 50 most influential articles on stroke rehabilitation: a bibliometric analysis. Am. J. Phys. Med. Rehabilitation, 10–1097. 10.1097/PHM.0000000000002412 38207163

[B46] Pennycott A. Wyss D. Vallery H. Klamroth-Marganska V. Riener R. (2012). Towards more effective robotic gait training for stroke rehabilitation: a review. J. neuroengineering rehabilitation 9 (1), 65–13. 10.1186/1743-0003-9-65 PMC3481425 22953989

[B47] Pinto-Fernandez D. Torricelli D. del Carmen Sanchez-Villamanan M. Aller F. Mombaur K. Conti R. (2020). Performance evaluation of lower limb exoskeletons: a systematic review. IEEE Trans. Neural Syst. Rehabilitation Eng. 28 (7), 1573–1583. 10.1109/tnsre.2020.2989481 32634096

[B48] Plummer P. Behrman A. L. Duncan P. W. Spigel P. Saracino D. Martin J. (2007). Effects of stroke severity and training duration on locomotor recovery after stroke: a pilot study. Neurorehabilitation neural repair 21 (2), 137–151. 10.1177/1545968306295559 17312089

[B49] Pons J. L. (2008). Wearable robots: biomechatronic exoskeletons. USA: John Wiley & Sons.

[B50] Postol N. Grissell J. McHugh C. Bivard A. Spratt N. J. Marquez J. (2021). Effects of therapy with a free-standing robotic exoskeleton on motor function and other health indicators in people with severe mobility impairment due to chronic stroke: a quasi-controlled study. J. Rehabilitation Assistive Technol. Eng. 8, 205566832110458. 10.1177/20556683211045837 PMC8543702 34707883

[B51] Pournajaf S. Calabrò R. S. Naro A. Goffredo M. Aprile I. Tamburella F. (2023). Robotic versus conventional Overground gait training in subacute stroke survivors: a multicenter controlled clinical trial. J. Clin. Med. 12 (2), 439. 10.3390/jcm12020439 36675371 PMC9861649

[B52] Rajasekaran V. López-Larraz E. Trincado-Alonso F. Aranda J. Montesano L. Del-Ama A. J. (2018). Volition-adaptive control for gait training using wearable exoskeleton: preliminary tests with incomplete spinal cord injury individuals. J. neuroengineering rehabilitation 15 (1), 4–15. 10.1186/s12984-017-0345-8 PMC5751847 29298691

[B54] Riener R. Nef T. Colombo G. (2005). Robot-aided neurorehabilitation of the upper extremities. Med. Biol. Eng. Comput. 43, 2–10. 10.1007/bf02345116 15742713

[B55] Rodríguez-Fernández A. Lobo-Prat J. Font-Llagunes J. M. (2021). Systematic review on wearable lower-limb exoskeletons for gait training in neuromuscular impairments. J. neuroengineering rehabilitation 18 (1), 22–21. 10.1186/s12984-021-00815-5 PMC7852187 33526065

[B56] Sanchez-Villamañan M. D. C. Gonzalez-Vargas J. Torricelli D. Moreno J. C. Pons J. L. (2019). Compliant lower limb exoskeletons: a comprehensive review on mechanical design principles. J. neuroengineering rehabilitation 16 (1), 55–16. 10.1186/s12984-019-0517-9 PMC6506961 31072370

[B57] Tefertiller C. Hays K. Jones J. Jayaraman A. Hartigan C. Bushnik T. (2018). Initial outcomes from a multicenter study utilizing the indego powered exoskeleton in spinal cord injury. Top. spinal cord Inj. rehabilitation 24 (1), 78–85. 10.1310/sci17-00014 PMC5791927 29434463

[B70] Tian D. Li F. He Y. (2024). Data-driven estimation for uphill continuous rehabilitation motion at different slopes using sEMG. Biomed Signal Processi and Con 93 (5), 106162.

[B58] Uivarosan D. Bungau S. G. Nistor-Cseppento C. D. Negru P. A. Bungau A. F. Sabau A. M. (2022). Application of robotic recovery techniques to stroke survivors—bibliometric analysis. J. Personalized Med. 12 (12), 2066. 10.3390/jpm12122066 PMC9788322 36556286

[B59] Vaughan-Graham J. Brooks D. Rose L. Nejat G. Pons J. Patterson K. (2020). Exoskeleton use in post-stroke gait rehabilitation: a qualitative study of the perspectives of persons post-stroke and physiotherapists. J. neuroengineering rehabilitation 17 (1), 123–215. 10.1186/s12984-020-00750-x PMC7488039 32912215

[B60] Warutkar V. Dadgal R. Mangulkar U. R. (2022). Use of robotics in gait rehabilitation following stroke: a review. Cureus 14 (11), e31075. 10.7759/cureus.31075 36475123 PMC9719588

[B61] Wu A. R. (2021). Human biomechanics perspective on robotics for gait assistance: challenges and potential solutions. Proc. R. Soc. B 288 (1956), 20211197. 10.1098/rspb.2021.1197 PMC8334844 34344175

[B62] Wu D. Zhang H. Leng Y. Li K. Li S. Lo W. L. A. (2023). A bibliometric analysis of telerehabilitation services for patients with stroke. Front. neurology 13, 1026867. 10.3389/fneur.2022.1026867 PMC9868953 36698904

[B63] Wu S. Wu B. O. Liu M. Chen Z. Wang W. Anderson C. S. (2019). Stroke in China: advances and challenges in epidemiology, prevention, and management. Lancet Neurology 18 (4), 394–405. 10.1016/s1474-4422(18)30500-3 30878104

[B64] Xu F. Bai L. Dai Z. Cheng H. (2023). Research hotspots and trends in post-stroke dysphagia: a bibliometric analysis. Front. Neurosci. 17, 1275748. 10.3389/fnins.2023.1275748 37942140 PMC10628302

[B65] Yang J. Gong Y. Yu L. Peng L. Cui Y. Huang H. (2023). Effect of exoskeleton robot-assisted training on gait function in chronic stroke survivors: a systematic review of randomised controlled trials. BMJ open 13 (9), e074481. 10.1136/bmjopen-2023-074481 PMC10503387 37709309

[B66] Yi X. Yubing D. (2024). Exoskeleton-assisted walking rehabilitation for spinal cord injury: CiteSpace analysis of research hotspots. Res. Chin. Organ. Eng., 5403–5412. 10.12307/2024.674

[B67] Young A. J. Ferris D. P. (2016). State of the art and future directions for lower limb robotic exoskeletons. IEEE Trans. Neural Syst. Rehabilitation Eng. 25 (2), 171–182. 10.1109/tnsre.2016.2521160 26829794

[B68] Yu D. Xu Z. Pedrycz W. Wang W. (2017). Information sciences 1968–2016: a retrospective analysis with text mining and bibliometric. Inf. Sci. 418, 619–634. 10.1016/j.ins.2017.08.031

[B69] Zuccon G. Lenzo B. Bottin M. Rosati G. (2022). Rehabilitation robotics after stroke: a bibliometric literature review. Expert Rev. Med. Devices 19 (5), 405–421. 10.1080/17434440.2022.2096438 35786139

